# A systematic review on how to treat deltoid ligament injuries - are we missing a uniform standard?

**DOI:** 10.1186/s12891-026-09660-w

**Published:** 2026-03-03

**Authors:** Nasef Mohamed N. Abdelatif, Judith Schrempf, Tim Schepers, Wolfgang Böcker, Hans Polzer, Sebastian Felix Baumbach

**Affiliations:** 1 Haed of Orthopedic Foot and Ankle Department, OrthoClinic, Private Practice, Cairo, Egypt; 2 Musculoskeletal University Centre Munich (MUM), University Hospital, Ludwig-Maximilians-University Munich (LMU), Munich, Germany; 3 OrthoPlus Munich, Munich, Germany; 4 Trauma Unit, Department of Surgery, Amsterdam UMC (Location AMC), University of Amsterdam, Amsterdam, The Netherlands

**Keywords:** Deltoid ligament injury, Deltoid ligament treatment, Deltoid ligament rupture, Deltoid ligament repair, Medial ankle instability, Collateral ligaments, Systematic review, Ankle joint

## Abstract

**Background:**

There is an vivid, ongoing discussion on whether injuries to the deltoid ligament complex, especially in the setting of ankle fractures, neccessitate surgical treatment. One reason for the conflicting results in literature, could be a missing standard on how acute deltoid ligament injuries are treated. The aim of this systematic review was to analyze the different applied treatment stratagies in studies reporting on the outcome of surgically treated acute deltoid ligament injuries.

**Methods:**

The herein conducted systematic review was conducted per PRISMA guidelines, the inclusion criteria were framed according to the PICOS criteria. The study was a-priori registered. Three independent reviewers conducted the literature search and data extraction (JS, AMN). The data assessed were study type, level of evidence, included fractures, methods of treating deltoid ligament injuries, differentiation between the superficial and deep layers and associated syndesmotic injuries.

**Results:**

Per the therapeutic studies (*n* = 37), the deltoid ligament repair was mostly conducted using suture anchors (*n* = 29), either placed in the medial malleolus for superficial deltoid ligament (SDL, *n* = 11)/ deep deltoid ligament (DDL, *n* = 7) repair and/ or in the medial talus for DDL repair (*n* = 10). 10 studies used direct sutures for SDL repair (*n* = 10) and/ or the DDL repair (*n* = 1). One study each used either a tibialis anterior tendon graft, a temporary arthrodesis of the ankle joint, or augmented the deltoid ligament.

**Conclusion:**

A missing standard for treating injuries to the deltoid ligament complex was observed. Although most studies used suture anchors, there is a huge heterogeneity regarding the placement, the number of anchors used for repair, and the exact layer(s) that were adressed.

Further research is needed to establish evidence-based guidelines on how to treat acute deltoid ligament injuries.

**Supplementary Information:**

The online version contains supplementary material available at 10.1186/s12891-026-09660-w.

## Introduction

The ankle joint is predominantly stabilized by its bony geometry, in addition to the syndesmotic and the deltoid ligament complex. The syndesmotic complex resembles a three-point suspension between the tibia and fibula, stabilizing the bony ankle mortise [3, 62]. The deltoid ligament complex comprises of a superficial (SDL) and deep (DDL) layer. It is believed to center the talus underneath the tibia and prevent medial talar translation [8, 17, 42, 46].

There is an vivid, ongoing discussion on whether injuries to the deltoid ligament complex, especially in the setting of ankle fractures, neccessitate surgical treatment [14, 20, 59]. One can find as many studies showing no benefit for an additional medial stabilization as for the superiority, if the deltoid ligament complex is repaired [14, 29, 59]. One reason for these inconclusive results could be a missing standard for the diagnosis and treatment of deltoid ligament injuries [48].

The diagnostic heterogeneity has been analyzed in a previous paper [48]. In the current paper the authors focus on the variability in treatment regimens. Possible treatment approaches range from non-operative treatment to direct sutures or single / double suture anchors [26, 30, 63, 68]. A non-standardized treatment bears the risk to result in medial ankle instability which might progress into flatfoot deformity or ankle osteoarthritis.

Various systematic reviews have tried to evaluate whether deltoid ligament repair does result in a better patient rated outcome [14, 20, 51, 59]. A recent narrative review assessed the development of deltoid ligament repair over a period of 4 decades. The authors found a trend towards deltoid ligament stabilization in more recent studies with favorable outcomes [55, 57]. Yet, no study has taken a step back and tried to systematically evaluate the various treatment strategies applied for deltoid ligament injuries. One can assume, that in case of considerably varying treatment approaches, the comparability of these studies is limited and therefore one cannot draw a summative conclusion from these studies.

The aim of the current study was to systematically analyze the therapeutic strategies for acute deltoid ligament injuries applied in outcome studies on deltoid ligament injuries.

## Material and method

The current systematic review was published a-priori to Prospero (Prospero ID: CRD42022307112) and followed the Preferred Reporting Items for Systematic Reviews (PRISMA) guidelines [41]. Due to a growing number of systematic reviews focusing on the patient rated outcome, the initial Prospero protocol was adapted. The systematic review consequently focused on the applied treatment approaches for deltoid ligament injuries.

### Search strategy, study selection, and data extraction

The search strategy and eligibility criteria were similar to a previous systematic review [48]. In brief, the search strategy (Supplement 1) combined the concepts of “Ligament” AND “Deltoid” AND “Injury / Rupture / Imaging / Diagnosis” and was applied to four common databases (MEDLINE (PubMed), Scopus, Central, and EMBASE), included a grey literature search, and was applied from inception to November 2024. All reference lists of papers included and excluded systematic reviews were hand-searched for further eligible studies.

Duplicates were removed in Endnote™ (Vs. 20.1; Fa. Clarivate) and the final dataset was imported into Covidence™ (Melbourne, Australia). Three independent reviewers (JS, ANMN; SFB resolved conflicts) performed the further study selectin process within Covidence™. Eligibility criteria were designed per the PICOS criteria. Eligible were studies on skeletal mature patients with an acute (or provoked) injury to the deltoid ligament complex, either isolated or in combination with an ankle fracture or syndesmotic injury. Studies must have been comparative and report on any objective outcome parameters. Eligible were any cadaver/biomechanical or clinical studies, regardless of the study design, with at least 10 patients included. Case reports, non-comparative studies and studies missing objective outcome parameter were excluded.

The data assessed were study type (e.g., case series, retrospective cohort, randomized clinical trial), the level of evidence, included fractures, differentiation between superficial and deep layers, methods of repair, suture anchor position, and associated syndesmotic injuries. Disagreement between the reviewers assessing the data was resolved by discussion (JS, ANMN, SFB).

### Risk of bias assessment

For each study, the level of evidence (criteria published by Wright et al. [64]), and the risk of bias were assessed. For clinical studies, the Methodological Index for Non-randomized Studies (MINORS) [3] was used, for cadaveric studies, the Quality Appraisal for Cadaveric Studies (QUACS) [61].

### Data analysis

A descriptive analysis was performed for the different treatment strategies applied per the addressed layer(s). These were listed separately by the method of repair, i.e. suture anchors, direct suture, and tendon graft. The number and placement of suture anchors were also assessed.

## Results

**Fig. 1:**
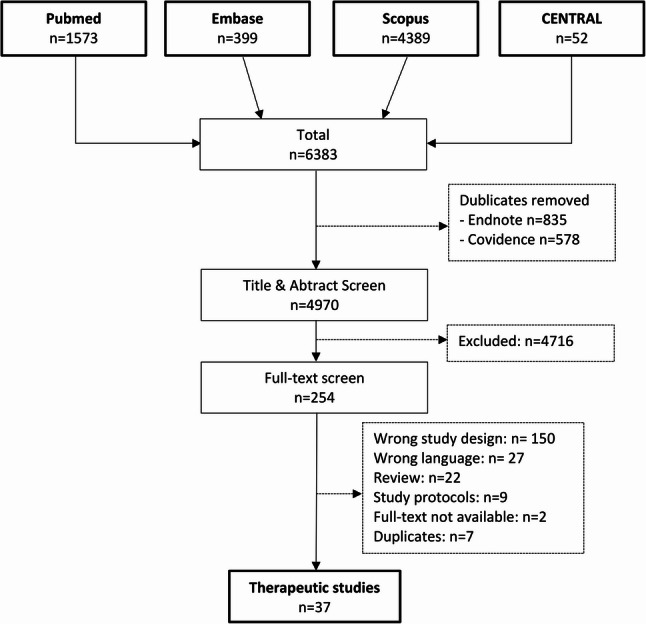
Study selection flow chart according to the PRISMA guidelines. n: numbers

As recommended by the PRISMA guidelines, the study selection process is summarized in Fig. 1. Overall, 4970 abstracts were screened, with 37 studies finally meeting the inclusion criteria (32 clinical and five biomechanical studies).

### Risk of bias assessment

The results of the risk of bias assessment are illustrated in Supplement 2. 7 studies were non-randomized, non-comparative studies with an average MINORS score of 8/16 [2, 16, 26, 36, 44, 50, 67]. 20 studies were comparative studies with a score of 13/24 [1, 9–11, 15, 31–35, 37, 38, 43, 52, 58, 60, 63, 66, 69, 70] and five studies were RCTs with 15/24 points on average [18, 45, 53, 54, 65]. For the five cadaveric studies the average QUACS score was 10/13 [6, 7, 21, 39, 47].

### Treatment strategies

#### What did the studies compare

Overall, eight studies compared two surgical treatment strategies [2, 6, 16, 26, 36, 44, 50, 67], five studies compared trans-syndesmotic fixation with deltoid ligament repair [31, 37, 45, 60, 65], two studies compared syndesmotic fixation and deltoid ligament repair with syndesmotic fixation alone [11, 38]. 15 studies compared a surgical strategy to a conservative treatment [1, 9, 10, 15, 18, 32, 33, 35, 52, 53, 58, 63, 66, 69, 70]. Three biomechanical studies compared the stability of the intact deltoid ligament with the stability achieved after various fixation methods for induced deltoid ligament injuries [7, 21, 47]. One study compared repair of the deep layer, the superficial layer and no repair of DL [54]. One study compared syndesmotic fixation and deltoid ligament repair and both, syndesmotic and deltoid ligament repair [39]. One study compared DL repair by placing suture anchors either at the medial Malleolus or the talus [43]. One study compared transarticular external fixation with deltoid ligament repair [34]. A detailed overview over these studies is given in Supplement 2.

#### Differentiation between the different DL layers

Out of the 37 studies included, 16 (43%) studies did not differentiate between the layers of the deltoid ligament complex [1, 7, 11, 15, 16, 18, 21, 26, 31, 32, 38, 39, 47, 50, 53, 60]. The remaining 21 (57%) studies did differentiate between the SDL and DDL [2, 6, 9, 10, 28, 33–37, 43, 44, 52, 54, 58, 63, 65–67, 69, 70]. Per the general surgical strategy, 16 studies (43%) addressed both layers [6, 10, 21, 28, 34, 36, 37, 39, 43, 47, 54, 63, 65, 67, 69, 70], six studies (16%) only addressed the SDL [1, 26, 33, 35, 44, 58], four (11%) only the DDL [15, 16, 52, 66], and the remaining 11 studies (30%) did not state, which layer was addressed [2, 7, 9, 11, 18, 31, 32, 38, 50, 53, 60].

#### General repair strategy

The deltoid ligament repair was conducted by suture anchors in 29 (78%) studies [2, 6, 10, 11, 16, 18, 26, 28, 31, 33–39, 43, 44, 47, 50, 52, 54, 58, 60, 63, 65, 67, 69, 70]. Additional procedures were performed in 11 of these studies in terms of additional direct sutures [10, 11, 28, 36, 37, 60, 63, 65, 67, 69, 70] and in one study by an additional ligament augmentation [6]. The remaining eight studies used direct sutures (*n* = 4) [1, 7, 15, 53], a tibialis anterior tendon graft (*n* = 1) [21], a temporary arthrodesis by tibiotalar inserted K-wires (*n* = 1) [66], or did not state the method of DL repair (*n* = 2) [9, 32].

#### Technique of repair per the DL-layers

**Fig. 2:**
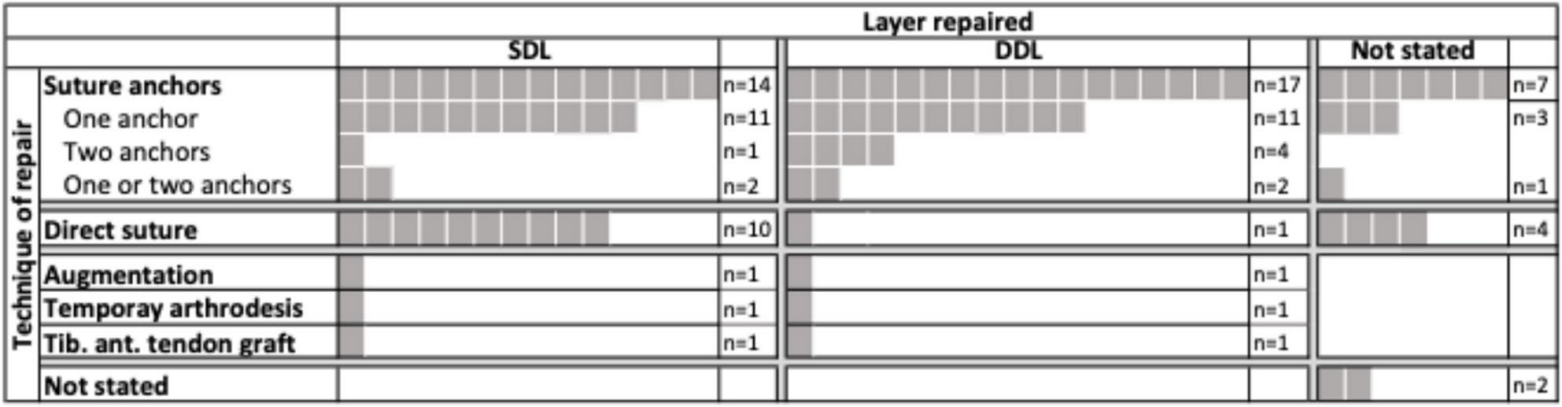
Repair technique per addressed layer. SDL: Superficial deltoid ligament; DDL: Deep deltoid ligament; n=numbers

Figure 2 depicts the repair technique applied per the DL-layers addressed. Each grey square resembles a study in which a therapeutic was applied as part of their management to address the deltoid ligament injury. If a single study applied several methods of management, i.e. direct suture and suture anchor(s), it is represented by several grey squares. The number of squares therefore do not resemble the number of studies, but the number of therapeutic methods applied. 16 studies addressed both, the SDL and DDL. Of these, nine used solely suture anchors [6, 28, 34, 36, 39, 43, 47, 54, 67], six studies used suture anchor(s) for DDL and direct sutures for SDL repair [10, 36, 37, 63, 65, 69, 70] and on study a tendon graft for DDL and SDL repair [21].

#### Anchor placement

**Fig. 3:**
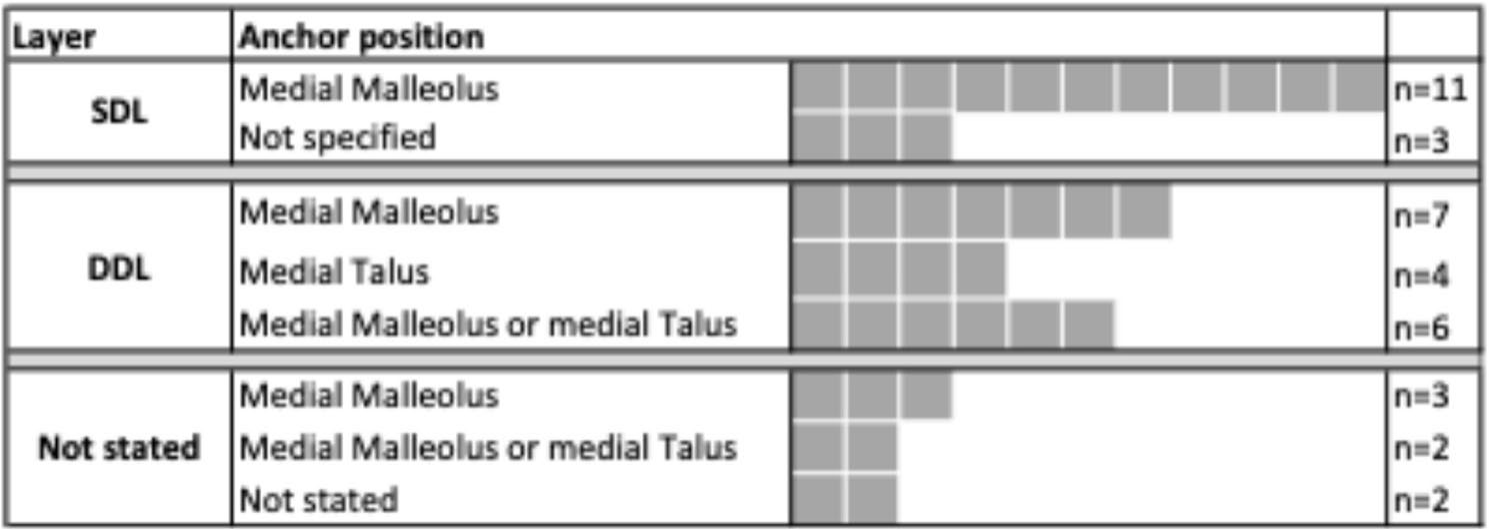
Anchor position per the addressed layer. SDL: Superficial deltoid ligament; DDL: Deep deltoid ligament; n=numbers

Figure 3 depicts the actual suture anchor position per the DL-layers addressed. The design of the figure is similar to Fig. 2 and it is based on those 29 studies using suture anchors for DL repair. For SDL repair, the suture anchors were placed in the medial malleolus in 11 studies [6, 21, 26, 34–36, 39, 43, 44, 47, 54]. The remaining three studies did not state where the anchors were placed [28, 33, 67]. For DDL repair, seven studies [6, 16, 28, 39, 47, 52, 69] placed suture anchors in the medial malleolus, and four studies [36, 37, 54, 67] in the medial talus. In six studies the anchors were placed in the medial malleolus or the medial talus for DDL repair [10, 34, 43, 63, 65, 67].

## Discussion

A recent narrative review pointed to a trend towards DL repair in more recent studies with a possible superiority for the patient rated outcome. Still, the available body of evidence remains inconclusive. One reason for these inconclusive findings could be varying surgical treatment strategies. The current systematic review found that 73% of the studies did either not differentiate between the different layers of the deltoid ligament (43%) or did not state which DL layer was addressed (30%). Of those studies differentiating between the DL layers, again almost half of the studies addressed both layers, 16%/11% addressed the SDL/DDL only. With respect to the actual repair strategy, the SDL was almost equilaterally repaired by using one suture anchor or direct sutures. The suture anchor was, when specified, always placed in the medial malleolus. The DDL was predominantly repaired by one suture anchor, which was placed either in the medial malleolus or talus.

The predominant rational for repairing the DL complex is to restore ankle anatomy and stability. Older studies focused their line of argumentation on restoring ankle anatomy [55]. As most studies reporting on DL-repair are ankle fracture studies, it appears reasonable, that anatomy restoration is predominantly achieved by open reduction and internal fixation of the fractures. Therefore, the authors of the earlier studies deemed DL-repair to be not necessary in the setting of an ankle fracture. More recent studies have additionally focused on ankle [21] /midfoot stability [40] and the actual long-term outcome. These studies, especially non-comparative studies, point towards a possible superiority of DL repair, possibly because of a better restauration of ankle stability. Still even today, comparative studies paint a more inconclusive picture. These inconclusive findings could very well be explained by the great heterogeneity observed in DL treatment strategies.

It appears astonishing to the authors, that most studies analyzed herein did either not differentiate between the DL layers addressed or did not state which layer was addressed. The SDL predominantly supports medial rotational stability and the DDL stabilizes the talus against posterior, lateral, and valgus movements [8, 12]. It might therefore not be surprising, that biomechanical studies were able to show that both, SDL and DDL, play an equal important role in ankle stability [22]. Consequently, the authors believe that distinguishing between the different DL layers is essential to guide the treatment. Future studies should meticulously state which layers were injured and which were consecutively addressed.

The SDL was almost equilaterally repaired by using one suture anchor or direct sutures. The suture anchor was, when specified, always placed in the medial malleolus. The actual treatment strategy is obviously guided by the type of SDL rupture. The SDL can either tear off or rupture close to the medial malleolus or in mid-substance [13, 24, 25, 63]. In case the SDL is torn of the medial malleolus, it can only be fixed by trans-osseous sutures or suture anchors. A mid-substance tear can more easily be addressed by direct sutures. The same stays true for the DDL, which most often either tears of the medial malleolus or the talus [25, 49, 63]. Mid-substance tears of the DDL are less frequent. DDL treatment, compared to SDL repair, is complicated by the limited space available and the smaller length dimension of the DDL. This might explain, why the working horse for DDL repair was the suture anchor.

The authors initially aimed to analyze the actual anchor position, especially at the medial malleolus, in more details. In theory, the anchor for the SLD could be placed in the anterior aspect of the anterior colliculus or anywhere in the anterior facet of the medial malleolus. The DDL anchor could be placed in the anterior- or posterior colliculus, or in the groove in-between. Previous biomechanical [57] and clinical studies [4, 43, 63] have assessed the effect of different anchor positions and the number of anchors used to stabilize the deltoid ligament. The findings are inconclusive and do not allow for a general recommendation. Not surprisingly, the studies analyzed herein were missing sufficient detail to assess the anchor position in more detail. Previous studies should not only specify which DL layer is addressed, but also the exact anchor location and suture technique used.

As outlined in the introduction, overall ankle stability is based on the bony integrity of the ankle joint, tibio-fibula, i.e. syndesmotic, mortis stability and medial ligamentous stability. Interestingly, some authors believe that syndesmotic- and medial side stability can counteractively compensate for each other. Therefore, there are surgeons who advocate, that a stabilization of the syndesmosis complex also restores medial side stability [65], or a stabilization of the medial side restores syndesmotic stability [23]. But the syndesmotic complex stabilizes the bony ankle mortise [3, 62]. The deltoid ligament complex centers the talus underneath the tibia and prevents medial talar rotation [8, 17, 42, 46]. As the syndesmotic and deltoid ligament complexes serve different biomechanical purposes, the authors believe, that they both therefore should be addressed. This could also be shown in biomechanical studies, which have highlighted the necessity of restoring both, syndesmotic- and DL to regain overall ankle rotational stability [22, 27, 39]. Especially in acute complex injuries of the ankle joint with suspected cartilage damage arthroscopically assisted techniques gain popularity these days [5]. More recent studies show that both, the superficial and deep layers of the deltoid can be adequately addressed arthroscopically [19, 56]. As a less invasive procedure this could be a chance to aim satisfying clinical outcome with faster recovery in medial ankle instability caused by deltoid ligament injuries (acute and chronic). Further clinical studies are in need to develop algorithms for the best clinical practice.

## Limitation of the study

Limitations of the herein study are based on the heterogeneity of the included studies. The considerable variability in study designs (e.g. cadaveric, clinical, comparative and non-comparative), surgical techniques, and associated fixation approaches (e.g. with or without syndesmotic fixation) challenges direct comparison and categorization. Additionally, many studies do not clearly specify which layers of the deltoid ligament were assessed or the exact surgical technique used.

## Strengths of the study

Thy study provides a comprehensive analysis of deltoid ligament repair strategies, highlighting the considerable variability in surgical approaches. Through an extensive search across multiple databases, it offers a structured evaluation of treatment techniques. It differentiates between the layers of the deltoid ligament and various treatment methods, including suture anchors, direct sutures, and anchor placement.

## Conclusion

The management of deltoid ligament injuries and instability is still missing a consensus. Literature is lacking exact definitions and evidence on how to treat deltoid ligament injuries. Most studies analyzed in the herein systematic review used one or two suture anchors for DDL-repair and one suture anchor or direct suture for SDL-repair. The superior method of fixation and position of SDL- and DDL-repair of this injury is yet to be settled upon. Many studies did not specify which layers of the deltoid ligament were surgically addressed or did not differentiate between the layers. This might be one reason for the variety of surgical strategies for deltoid ligament reconstruction. Evidently high-level randomized trials in addition to adequately performed biomechanical studies are in abundant demand.

## Supplementary Information

Supplementary Material 1.

Supplementary Material 2.

Supplementary Material 3.

Supplementary Material 4.

Supplementary Material 5.

## Data Availability

All data generated or analyzed during this study are included in this published article and its supplementary information files.
