# Supplementary material for: A systematic review on how to treat deltoid ligament injuries - are we missing a uniform standard?
Source: BMC Musculoskelet Disord. 2026 Mar 3;27:287. doi: 10.1186/s12891-026-09660-w (PMC13063554; doi:10.1186/s12891-026-09660-w)
Supplement: Supplementary file 1 — Supplementary Material 1. [file 12891_2026_9660_MOESM1_ESM.pdf]

Search strategy

PubMed (Medline)

("Ligaments"[MESH] OR "Ligament*"[tiab]) AND ("Deltoid*" OR ("Medial"[tiab] AND ("Ankle"[tiab] OR "Spring"[tiab] OR "tender*"[tiab]))) AND ("Wounds and Injuries"[Mesh] OR "Rupture"[Mesh] OR "Injur*"[tiab] OR "rupture*"[tiab] OR "Joint Instability"[Mesh] OR "Instability"[Mesh] OR "Imag*"[tiab] OR "Diagno*"[tiab] OR "Stress, Mechanical"[Mesh] OR "Ankle Joint/diagnostic imaging"[MAJR] OR "Stress"[tiab] OR "test"[tiab])

Embase/ Ovid

(ligament.ab,kf,ti OR ligament*.ab,kf,ti) AND (Deltoid*.ab,kf,ti OR (medial collateral ligament.ab,kf,ti AND (Ankle.ab,kf,ti OR Spring.ab,kf,ti OR tender*.ab,kf,ti))) AND (Wound.mp OR Injury.mp OR Rupture.mp OR Injur*.ab,kf,ti OR rupture*.ab,kf,ti OR (Joint.mp AND Instability.mp) OR Instability.mp OR Imag*.ab,kf,ti OR Diagno*.ab,kf,ti OR Stress.mp OR Mechanical.mp OR (Ankle.mp AND Joint.mp AND diagnostic.mp AND imaging.mp OR Stress.ab,kf,ti OR test.ab,kf,ti))

Scopus

("Ligaments" OR TITLE-ABS("Ligament*")) AND ("Deltoid*" OR (TITLE-ABS("Medial") AND (TITLE-ABS("Ankle") OR TITLE-ABS("Spring") OR TITLE-ABS("tender*")))) AND (TITLE-ABS("Wounds and Injuries”) OR TITLE-ABS("Rupture") OR TITLE-ABS("Injur*") OR TITLE-ABS("rupture*") OR TITLE-ABS("Joint Instability") OR TITLE-ABS("Instability") OR TITLE-ABS("Imag*") OR TITLE-ABS("Diagno*") OR TITLE-ABS("Stress”) OR TITLE-ABS(“Mechanical") OR (TITLE-ABS("Ankle Joint”) AND TITLE-ABS(“diagnostic imaging")) OR TITLE-ABS("Stress") OR TITLE-ABS("test"))

Central

−	+	#1	MeSH descriptor: [Ligaments] explode all trees	MeSH	1209
−	+	#2	"ligament*":ti,ab,kw	Limits	5675
−	+	#3	"Deltoid*" OR ("Medial":ti,ab,kw AND ("Ankle":ti,ab,kw OR "Spring":ti,ab,kw OR "tender*":ti,ab,kw))	Limits	1783
−	+	#4	MeSH descriptor: [Wounds and Injuries] explode all trees	MeSH	27850
−	+	#5	MeSH descriptor: [Rupture] explode all trees	MeSH	1273
−	+	#6	"Injur*":ti,ab,kw OR "rupture*":ti,ab,kw	Limits	6276
−	+	#7	MeSH descriptor: [Joint Instability] explode all trees	MeSH	797
−	+	#8	"Instability":ti,ab,kw OR "Imag*":ti,ab,kw OR "Diagn*":ti,ab,kw	Limits	6602
−	+	#9	MeSH descriptor: [Stress, Mechanical] explode all trees	MeSH	1356
−	+	#10	MeSH descriptor: [Ankle Joint] explode all trees	MeSH	770
−	+	#11	MeSH descriptor: [Diagnostic Imaging] explode all trees	MeSH	50366
−	+	#12	"Stress":ti,ab,kw OR "test":ti,ab,kw	Limits	323180
−	+	#13	(#1 OR #2) AND (#3) AND (#4 OR #5 OR #6 OR #7 OR #8 OR #9 OR #10 OR #11 OR #12)	Limits	52
−	+	#14	Type a search term or use the S or MeSH buttons to compose S MeSH	Limits	N/A

Grey literature

Opengrey.eu, ntis.gov and the Healthcare Management Information Consortium (HMIC) was screened
