# Supplementary material for: A systematic review on how to treat deltoid ligament injuries - are we missing a uniform standard?
Source: BMC Musculoskelet Disord. 2026 Mar 3;27:287. doi: 10.1186/s12891-026-09660-w (PMC13063554; doi:10.1186/s12891-026-09660-w)
Supplement: Supplementary file 2 — Supplementary Material 2. [file 12891_2026_9660_MOESM2_ESM.pdf]

	**Objective stated** The study's aims are clearly stated. Hypotheses are presented	**Basic information about sample is included** Age (range or mean and standard deviation), gender and sample size are stated	**Applied methods are described comprehensibly** Clearly structured, detailed outline of the study protocol and the process of dissection	**Study reports condition of the examined specimens** Specimens' status (healthy vs. injured/with diseases, embalmed vs. fresh) is stated. In case of embalmed cadavers, type of solution is reported	**Education of dissecting researchers is stated** Study reports knowledge/professional state and/or experience of the investigator	**Findings are observed by more than one researcher** Stated clearly, that two or more persons independently made the observations	**Results presented thoroughly and precise** Results described with clear structure, and including, figures, illustrations or tables	**Statistical methods appropriate** If applicable: correct choice and application of statistical data analysis	**Details about consistency of findings are given** Information on number or percentage of cases the observation was made in (e.g. continuity in 5 of 10 specimens)	**Photographs of the observations are included** Photographs of the key observations (e.g. continuities) with precise labels are included	**Study is discussed within the context of the current evidence** Other relevant trials relating to the field of study are stated and discussed	**Clinical implications of the results are discussed** Similar studies are reported, added knowledge and its relevance to the field are pointed out	**Limitations of the study are addressed** Weaknesses and methodological shortcomings are reported	**Points**	**Average Result**
**Biomechanical Studies (n=5)**															**10/13**
Haddad,Steven (2010)	1	1	1	1	0	0	1	1	1	1	1	1	1	11/13	
Butler, Bennet (2020)	0	1	1	0	0	0	1	1	1	1	1	1	1	9/13	
Mococain, Pablo (2020)	1	1	1	0	0	0	1	1	1	1	1	1	1	10/13	
Schottel, PC (2016)	1	1	1	0	0	0	1	1	1	1	1	1	1	10/13	
Brady, Alex W (2023)	1	1	1	0	0	0	1	1	1	0	1	1	1	9/13	
