# Supplementary material for: A systematic review on how to treat deltoid ligament injuries - are we missing a uniform standard?
Source: BMC Musculoskelet Disord. 2026 Mar 3;27:287. doi: 10.1186/s12891-026-09660-w (PMC13063554; doi:10.1186/s12891-026-09660-w)
Supplement: Supplementary file 4 — Supplementary Material 4. [file 12891_2026_9660_MOESM4_ESM.pdf]

Included studies - General study information

Comparative studies (No Repair/ Syndesmotic Repair/ DL Repair)

Author (Year)	Study Design (Level of Evidence)	Quality Assesment (MINORS)	Included Fractures
Asadi, Kamran (2021)	Retrospective cohort study (LoE: III)	13/24	AO type 44-A: n=12<br>AO type 44-B: n=35<br>AO type 44-C: n=18
Chen, Hongfeng (2020)	Retrospective cohort study (LoE: III)	14/24	SER IV: n=63
Choi, SeongJu (2020)	Retrospective cohort study (LoE: III)	15/24	SER IV: n=34
Dagtas, Mirza Zafer (2021)	Retrospective cohort study (LoE: III)	12/24	Distal fibular fractures: n=78<br>44-B: n=38<br>44-C: n= 40
Gu, Guanxue (2017)	Randomized controlled Study (LoE: I)	15/24	Not stated (´ankle fractures´)
Li, Ting (2020)	Retrospective cohort study (LoE: III)	8/24	Distal fibular fractures: n=71<br>Weber B: n=42<br>Weber C: n= 29
Liao, Junyi (2022)	Retrospective cohort study (LoE: III)	14/24	Lateral malleolus fracture combined with deltoid ligament and inferior tibiofibular syndesmotic disruption: n=78<br>Weber B: n=27<br>Weber C: n=51
Rosa, Isabel (2019)	Randomized controlled Study (LoE: I)	15/24	Distal fibular fractures: n=65
Sogbein, Olawale (2021)	Retrospective cohort study (LoE: III)	14/24	Lateral malleolar fractures (bimalleolar equivalent ankle fractures): n=147<br>Weber B: n=93<br>Weber C: n=54
Stromsoe, Knut (1995)	Randomized controlled Study (LoE: I)	14/24	Distal fibular fractures: n=50<br>Weber B: n=30<br>Weber C: n=20
Sun, Xu (2018)	Randomized controlled Study (LoE: I)	13/24	Distal fibular fractures<br>Weber B: n=41
Wang, Xu (2017)	Prospective cohort study (LoE: II)	12/24	SER type IV: n=35
Whitlock, Keith (2022)	Prospective cohort study (LoE: II)	14/24	Isolated, unstable distal fibula fractures:<br>Weber B/C: n=108
Woo, Seung Hun (2017)	Retrospective cohort study (LoE: III)	16/24	Lateral malleolar fractures: n= 78<br>SER: n=62<br>PER: n=16
Yang, Tao (2023)	Retrospective cohort study (LoE: III)	14/24	Ankle fractures: n=46<br>SER IV: n=23<br>PER IV: n=17<br>PA III: n=6
Zhang, Le (2023)	Retrospective cohort study (LoE: III)	11/24	Ankle fractures: n=210
Zhao, Hong-Mou (2017)	Retrospective cohort study (LoE: III)	15/24	AO-type B (SER): n=49<br>AO-type C (PER/PA): n=25
**All repaired studies**			
Diab, Hossam (2017)	Prospective case series (LoE: IV)	8/16	Distal fibular fractures: n=27
Hsu, Andrew (2015)	Retrospective case series (LoE: IV)	7/16	Not stated (´high-energy ankle fractures´): n=14
Liang, Wei (2023)	Retrospective case series (LoE: IV)	10/16	Ankle fractures: n=64
Mansur, Nacime (2021)	Retrospective case series (LoE: IV)	8/16	Ankle fractures: n=20
Rigby, Rian (2023)	Retrospective case series (LoE: IV)	6/16	Ankle fractures: n=47<br>Weber A: n=1<br>Weber B: n=19<br>Weber C +/- posterior malleolar fracture: n=17<br>Maissoneuve fracture: n=6<br>Medial malleolar avulsion: n=2<br>Bimalleolar fracture: n=3
Shen, Jian-Jian (2019)	Prospective case series (LoE: IV)	7/16	44-B: n=25<br>44-C: n=9<br>SER IV: n=11<br>PER III/IV: n=21<br>PA III: n=2
Yu, Guang-rong (2015)	Prospective case series (LoE: IV)	10/16	43-B: n=60<br>44-C: n=71<br>SERIV: n=42<br>PER III/IV: n=84<br>PA III: n=5
**Other**			
Baird, Robert (1987)	Retrospective cohort study (LoE: III)	10/24	SER IV: n=13<br>PER III/IV: n=11
Chen, Pei-Yu (2008)	Prospective cohort study (LoE: II)	13/24	Weber B: n=15
De Souza, L.J. (1985)	Retrospective cohort study (LoE: III)	9/24	Weber B: n=12<br>Weber C: n= 10
Jones, Christopher (2015)	Retrospective cohort study (LoE: III)	13/24	Isolated SER IV/ bimalleolar equivalent fractures: n=27
Lee, Tae Hoon (2016)	Prospective cohort study (LoE: II)	11/24	Isolated lateral malleolar fractures: n=35
Li, Bohua (2019)	Retrospective cohort study (LoE: III)	14/24	44-B3.1 (SER IV): n=23
Wu, Kai (2017)	Retrospective cohort study (LoE: III)	16/24	Weber B: n=19<br>Weber C: n=32<br>SER: n=22<br>PER: n=18<br>PAR: n=11
**Biomechanical studies**			
		**Qualitiy Assesment (QUACS)**	
Haddad Steven (2010)		11/13	No
Butler Bennet (2020)		9/13	SER IV
Mococain Pablo (2020)		10/13	No
Schottel PC (2016)		10/13	No
