# Supplementary material for: A systematic review on how to treat deltoid ligament injuries - are we missing a uniform standard?
Source: BMC Musculoskelet Disord. 2026 Mar 3;27:287. doi: 10.1186/s12891-026-09660-w (PMC13063554; doi:10.1186/s12891-026-09660-w)
Supplement: Supplementary file 5 — Supplementary Material 5. [file 12891_2026_9660_MOESM5_ESM.pdf]

**Included studies - Deltoid Repair**									
**Comparative studies (No Repair/ Syndesmotic Repair/ DL Repair)**									
**Author (Year)**	**Differentiation SDL/DDL**	**Layer addressed**	**Repair method SDL**	**Repair method DDL**	**Repair method layer n/a**	**Suture anchor position SDL**	**Suture anchor position DDL**	**Suture anchor position Layer n/a**	**Differentiation syndesmotic / deltoid injuries**
Asadi, Kamran (2021)	No	n/a			n/a				No
Chen, Hongfeng (2020)	Yes	SDL + DDL	Direct repair	Two anchors			Medial Malleolus + Medial Talus		Yes
Choi, SeongJu (2020)	No	n/a			Direct repair + anchor (n/a)			Medial Malleolus	Yes
Dagtas, Mirza Zafer (2021)	No	n/a			One or two anchors			Medial Malleolus or medial Talus	Yes
Gu, Guanxue (2017)	No	n/a			Anchor (n/a)			n/a	No
Li, Ting (2020)	Yes	SDL	One anchor			Tip of medial Malleolus			Yes
Liao, Junyi (2022)	Yes	SDL + DDL	Direct repair	One anchor			Medial Talus		Yes
Rosa, Isabel (2019)	Yes	SDL + DDL	One Anchor + direct repair	One anchor		n/a	Medial Malleolus		Yes
Sogbein, Olawale (2021)	Yes	DDL		One anchor			Medial Malleolus		Yes
Stromsoe, Knut (1995)	No	n/a			Direct repair				No
Sun, Xu (2018)	Yes	Yes (Group I: DDL; Group II: SDL)	One anchor	One anchor		Tip of anterior colliculus			Yes
Wang, Xu (2017)	Yes	SDL	One anchor			10mm from the tip of the anterior colliculus			No
Whitlock, Keith (2022)	No	n/a			Anchor (n/a) + Direct repair			n/a	Yes
Woo, Seung Hun (2017)	Yes	SDL + DDL	Direct repair	One or two anchors			Medial Malleolus + Medial Talus		Yes
Yang, Tao (2023)	Yes	DDL		K-Wire arthrodesis			K-Wire Medial Malleolus		Yes
Zhang, Le (2023)	Yes	SDL + DDL	Direct repair	One anchor			Medial Malleolus		No
Zhao, Hong-Mou (2017)	Yes	SDL + DDL	Direct repair	One anchor			Medial Malleolus + Medial Talus		Yes
**All repaired studies**									
Diab, Hossam (2017)	No	DDL		One anchor			Medial Malleolus		Yes
Hsu, Andrew (2015)	No	SDL	One or two anchors			5mm above the tip of the medial Malleolus			Yes
Liang, Wei (2023)	Yes	SDL + DDL	One anchor + direct repair	One anchor		Anterior aspect of the medial Malleolus	Medial Talus		Yes
Mansur, Nacime (2021)	Yes	n/a			One anchor			n/a	Yes
Rigby, Rian (2023)	Yes	SDL	One or two anchors			Anterior and/or posterior colliculus			Yes
Shen, Jian-Jian (2019)	No	n/a			One anchor			Medial Malleolus or medial Talus	Yes
Yu, Guang-rong (2015)	Yes	SDL + DDL	One anchor + direct repair	One anchor		n/a	Medial Talus		No
**Other**									
Baird, Robert (1987)	No	SDL	Direct repair						Yes
Chen, Pei-Yu (2008)	Yes (sonography)	n/a			n/a				No
De Souza, L.J. (1985)	No	DDL		Direct repair					No
Jones, Christopher (2015)	No	n/a			One anchor			Medial Malleolus	No
Lee, Tae Hoon (2016)	Yes	SDL	One anchor			n/a			Yes
Li, Bohua (2019)	Yes	SDL + DDL	One anchor	One anchor		Medial Malleolus	Medial Malleolus + medial Talus		No
Wu, Kai (2017)	Yes	SDL +DDL	Direct repair	One anchor			Medial Malleolus + medial Talus		Yes
**Biomechanical studies**									
Haddad Steven (2010)	No	SDL + DDL	M. tibialis ant. tendon graft	M. tibialis ant. tendon graft					
Butler Bennet (2020)	No	n/a			Direct repair				
Mococain Pablo (2020)	No	SDL + DDL	Two anchors	Two anchors		Medial Malleolus	Medial Malleolus		
Schottel PC (2016)	No	SDL + DDL	One anchor	One anchor		Intercollicular groove	Medial Malleolus		
